# Miescher's granulomatosis (granulomatosis disciformis chronica et progressiva) in a non-diabetic patient – case report

**DOI:** 10.1186/1746-1596-4-28

**Published:** 2009-08-28

**Authors:** Beata Bergler-Czop, Ligia Brzezińska-Wcisło, Iwona Rogala-Poborska

**Affiliations:** 1 Department of Dermatology Silesian Medical University 40-027 Katowice Francuska Street 20/24 Poland; 2 42-624 Ossy ul. Leśna 2a Poland

## Abstract

**Introduction:**

Necrobiosis lipoidica diabeticorum is a rare disease of unclear etiology, that occurs in about 1% of diabetic patients.

**Case report:**

We present case of granulomatosis disciformis chronica et progressiva Miescher with good response to systemic corticosteroids therapy.

Patient 45 years old woman, with primary yellow-brown areas skin lesions, with foci well separated from surroundings on both lower legs, that occurred 5 years ago. In laboratory tests there was no abnormalities. Because of advance suggestion (after last admit in dermatological ward) of observation according to xantogranuloma necrobioticum tests for paraproteinemia were made. Immunoelectrophoresis, IgG, IgM, IgA levels, kappa light chain, lambda heavy chain; were correct, Bence-Johns protein-negative. During hospitalization in Clinic methylprednisolone in dose of 32 mg od, vascular drugs and local steroidotherapy was applied with good therapeutic response.

**Conclusion:**

We described case of typical clinical and histological characters of necrobiosis lipoidica. without diabetes-granulomatosis disciformis chronica et progressiva Miescher that despite of suspicion of proper diagnosis for a long time was not treat effective.

## Introduction

Necrobiosis lipoidica diabeticorum is a rare disease of unclear etiology that occurs in about 1% of diabetic patients [1]. The disease is characterized by a chronic inflammatory granulomatous process accompanied by vasculitis with perivascular deposits of complement C3 and immunoglobulins IgG, IgM and IgA. Typically found on the lower legs, focal skin lesions comprise well-separated irregular areas of discolored (yellow-brown) tissue. Tissue damage in the centre of foci includes atrophy, sclerosis and teleangiectasia [1-3].

Miescher's granulomatosis (also known as granulomatosis disciformis chronica et progressiva) was first reported by Miescher and Leder in 1948. In this condition skin lesions are typically localized bilaterally and symmetrically on the lower legs, and in the absence of diabetes [1,4]. Treatment of all types of necrobiosis lipoidica is difficult, and the condition can be unresponsive to systemic corticosteroids, cyclosporin A, retinoids, and antimalarial and vascular drugs. In contrast, some promising results have been obtained with phototherapy (psoralen and UVA treatment; PUVA), with topical application of corticosteroids or tacrolimus, or with photodynamic therapy [5-11].

We present a case of Miescher's granulomatosis without diabetes showing a favorable response to systemic corticosteroid therapy.

### Case report

**Figure 1:**
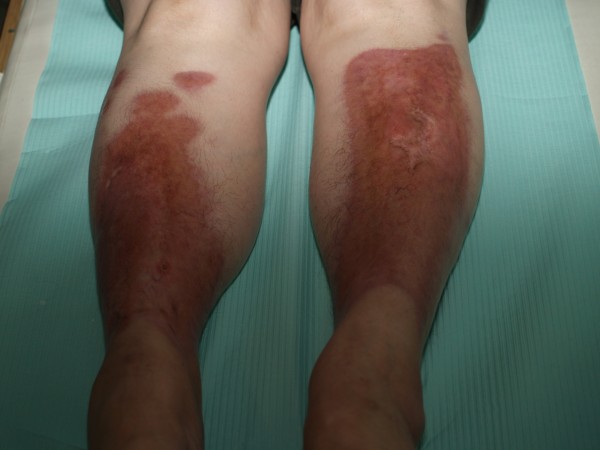
**The patient (female, 45 yr) with primary skin lesions consisting of discolored (yellow-brown) areas on both lower legs with well-separated foci**.

The patient (female, 45 yr) had primary skin lesions consisting of discolored (yellow-brown) areas on both lower legs with well-separated foci (Fig. 1). Her condition was first reported 5 years earlier. The initial diagnosis was erythema induratum, and treatment included penicillin G procaine, rifampicin, vascular drugs and local steroidotherapy. Diagnosis of Miescher's granulomatosis was suggested 3 years later on the basis of histopathologic examination and after exclusion of diabetes. We repeated penicillin G procaine, vascular drug administration and steroidotherapy. Cryotherapy with ethyl chloride was also applied. Despite the treatment the extent of the skin lesions increased slowly and these were accompanied by with small ulcerations within the lesion foci, often as a result of local injury.

Thyroid disease was diagnosed in 2007 (Hashimoto's thyroiditis; struma nodosa partim lymphomatosa Hashimoto) and subtotal resection of the thyroid gland was performed. Thyroid hormone replacement was with Eltroxin (levothyroxine, 100 mg). In the same year the patient was hospitalized in a provincial dermatological ward where a diagnosis of xanthogranuloma necrobioticum was suggested. Because treatment brought no improvement, and the extent of the skin lesions continued to increase, the patient was admitted to Department of Dermatology at Katowice Medical University.

Laboratory investigations included ESR 32/52, blood smear, electrolyte levels, AspAT, ALAT, GGTP, bilirubin, creatinine, urea, glucose, blood glucose profile, blood protein electrophoresis, CPK, aldolase, urine analysis, arthus-type reactions (latex-R, Waaler-Rose test, ASO). All were in the normal range. Because xanthogranuloma necrobioticum had been suggested tests for paraproteinemia were also performed. Immunoelectrophoresis revealed normal levels of IgG, IgM, IgA, kappa light chain, and lambda heavy chain; there was no evidence of Bence-Jones proteins.

In therapy methylprednisolone (32 mg od) was administered in conjunction with vascular drugs and local steroidotherapy. After 1 month of methylprednisolone there was significant improvement, with flattening and blanching of skin lesions accompanied by healing of minor ulcerations on the left lower leg. The patient is now receiving dermatologic out-patient care with continued administration of methylprednisolone and gradual dose reduction (table 1).

**Table 1:** Clinical history of the patient

date	disease	lab results	therapy	result (skin)
2002	erythema induratum (?)	normal range	penicillin G procaine, rifampicin, vascular drugs, local steroidotherapy	stabile

2005	Miescher's granulomatosis	histopathologic examination – Miescher's granulomatosis	penicillin G	stabile

2007	Hashimoto's thyroiditis	Anty-TPO antibodies – high range, T3, T4 – low range, TSH – high range, ultrasonography, histopathologic examination – Hashimoto's thyroiditis	subtotal resection of the thyroid gland Eltroxin (levothyroxine, 100 mg)	stabile

2007	xanthogranuloma necrobioticum (?)	normal range	micromolecular heparin vascular drugs	deterioration

2007	Miescher's granulomatosis	ESR 32/52, the rest – normal range	methylprednisolone (32 mg od)	improvment

## Discussion

Necrobiosis lipoidica in its classical form is a granulomatous disease of unclear etiology usually associated with diabetes. Ho et al. [12] reported on an atypical familial case where necrobiosis lipoidica without diabetes was found in 2 sisters. Flann et al. [13] and Criado et al. [14] described many histological similarities between the progression of xanthogranuloma necrobioticum and Miescher's granulomatosis, although the overall clinical picture differs between the two conditions. Leroy et al. [15] performed electron microscope ultrastructural studies in a patient (62 yr) with a diagnosis of necrobiosis lipoidica without diabetes. Here perivascular macrophage infiltration, necrobiosis and collagen fiber damage was reported but without loss of vascular wall integrity.

Treatment of classical variants of necrobiosis lipoidica and Miescher's granulomatosis is difficult. Bawaria et al. [16] applied pentoxifylline (3 × 400 mg td) in a 20-yr-old patient with diabetes and necrobiosis lipoidica. Tan et al. [17] used systemic corticosteroid with success in patients with necrobiosis lipoidica and insulin-dependent diabetes, and without destabilization of blood glucose levels. In our patient, long-term topical corticosteroid therapy gave no detectable clinical improvement.

Narbutt et al. [10] used local PUVA therapy (0.005% 8-methoxypsoralen followed by UVA irradiation) in 10 patients. After an average of 47 sessions (total dose 69.5 J/cm^2^) they observed recurrence of skin lesions in only 2 patients. Kreuter et al. [18] used fumaric acid esters (FAE) for the therapy of necrobiosis lipoidica, granuloma annulare and a skin variant of sarcoidosis. In 18 necrobiosis patients receiving FAE over 6 mo there was a significant improvement in skin status.

Bouhanick et al. [19] complemented steroid therapy with 113 sessions in hyperbaric chamber. This joint therapy was effective in a patient (28 yr) with insulin-dependent diabetes. Reinhard et al. [20] obtained good results in necrobiosis lipidica treatment using mycophenolate mofetil. Nguyen et al. [21] described an individual case of therapeutic success in a patient with necrobiosis after administration of an antimalarial drug (chloroquine). Owen et al. [22] reported a case of a 44-yr-old woman with ulcerated necrobiosis lipoidica that healed following grafting with dermal tissue engineered in culture. Zeichner et al. [23] treated necrobiosis lipoidica using a TNF inhibitor (etanercept).

Long-term risks associated with necrobiosis lipoidica include the development of spinocellular carcinoma. Tschuchnigg et al. [24] detected nodules within the necrobiosis foci in a 53-yr-old patient; these were diagnosed histologically as spinocellular carcinoma. Santos-Juanes et al. [25] made similar observations in a 75-yr-old patient with a 30 yr history of necrobiosis lipoidica.

## Conclusion

We have described a case with typical clinical and histological features of necrobiosis lipoidica in the absence of diabetes, also known as Miescher's granulomatosis. Treatment of this type of necrobiosis is difficult, and the condition failed to respond to different attempts at therapy over several years. We report that systemic steroidotherapy with methylprednisolone was of significant clinical benefit in this patient. Early diagnosis and treatment can not only improve the patient's quality of life but also protect against serious side-effects including spinocellular carcinoma.

## Consent

We confirm that written consent was obtained from the patient or their relatives for publication of study and the use of any images. A copy of the written consent in available for review by the Editor-in-Chief of this Journal.

## Competing interests

The authors declare that they have no competing interests.

## Authors' contributions

This manuscript was drafted by BBC, LBW, IRP. BBC – main conception, design, acquisition of data, interpretation of data, writing; LBW – references, writing assistance; IRP – writing assistance, acquisition of data All authors contributed to its critical review and all approved the final draft.
